# Treatment by Sleep and Suggestion
*Psycho-Therapeutics; or, Treatment by Sleep and Suggestion. By C. Lloyd Tuckey, M.D. Baillière, Tindal, and Cox, King William Street, Strand. 1889.


**Published:** 1889-03-23

**Authors:** 


### TREATMENT BY SLEEP AND SUGGESTION.*

DR. TUCKEY informs us that the system of psycho-therapeutics has attained its fullest development in Holland, and is also established in Germany, Russia, and Sweden. The system is thus described—When the patient's turn comes, "he is told to take his place in an arm-chair and to make his mind as much a blank as possible—to think of nothing at all—and to fix his eyes and attention on some special object; almost anything will do, from the operator's face or hand to a mark on the ceiling, or the pattern of the carpet. Then the phenomena which attend the on-coming of natural sleep are gradually suggested to him. 'Your sight is growing dim;' 'your eyelids are becoming heavy;' 'numbness is creeping over your limbs; you are getting more sleepy; you cannot keep your eyes open. Here the eyes close of themselves, or are closed by the operator, and it is generally found that the patient is indeed asleep." About two minutes of this talk about sleep, will, it is stated, usually produce the hypnotic effect on a new patient. The patient being thus influenced, the next treatment consists essentially in directing the invalids attention to the part affected, and suggesting an amelioration or disappearance of the morbid condition and symptons. If the malady is chronic, nervous headache for instance, the part of the head is gently rubbed, so that the patient's attention may be attracted to it, and he is told that the pain is to disappear, that he will awake feeling well, and that there will be no return. "This is generally enough," the patient is awaked and "goes about his work as usual."

Now there is nothing mysterious in this sleep. The patient, by fixing his attention on a certain point, strains the accommodation of the eyes, and strains the sight. The strain causes dilatation of the pupils and dimness of vision. The feeling of heaviness in the eyelids results from the fatigue of keeping them open in a strained position, and the assertion that the eyes are becoming tried, and the sight dim, is therefore founded on physiological data. The eyes being tired, the natural impulse is to close them, and this act calls up an association of ideas connected with fatigue, which points to sleep, aided by the monotonous tones of the operator suggesting it. It is contended that the hypnotic influence is not mesmerism, at least as exhibited by charlatans, because no unusual gifts are needed to practise the system. We must confess, however, that we do not recognize any difference between the condition of sleep now called hypnotism and mesmerism. A person composing himself and looking at the end of his nose will probably go to sleep, and if he remains long enough in such attitude, will attain towards that Nirvana which, by some Asiatics is regarded as the *summum bonum* of this world. A person staring at a disk of copper let into a piece of zinc, will probably go to sleep even without the chemical interchange which is said to take place between the two metals. In fact, if a person stares long enough at a piece of cork held on the palm of the hand, he will go to sleep. If a magnetiser makes "passes" over a persons eyes, the person will either go to sleep, or the muscles of the eyelids becoming tired by constantly following the movements of the operator's fingers, the victim will be unable to open his eyes. Much, however, depends on constitutional idiosyncracy, when one person essays to send another to the land of dreams The operater must at least assume a confidence, even if he has it not; and the person operated on must not be under any strong emotion, neither should he wish to resist the somnolent influence. Above all, it is desirable there should be a nervous or even hysterical temperament, although indeed this is desired by some professors. For instance, Professor Wettershand, as reported in the *British Medical Journal* last year, considers hysterical people not so susceptible, and states that after thirty years of age persons are less impressionable. We agree however with Professor Charcot, who asserts that hypnotion is suitable, and indeed possible only, for nervous and hysterical subjects. As with its predecessor mesmerism, so with hypnotism, it is nervous or hysterical persons who are most susceptible, and it is nervous or hysterical complaints which are said to be benefited. At page 25, Dr. Tuckey admits that severe pain will prevent the hypnotic sleep, yet we find cases of rhematism in the shoulder, articular rheumatism, neuralgia, sciatica, etc., mentioned as cured by hypnotism and suggestion. In other records we find that hysterical vomiting has been cured, and that an ingrowing toe-nail has been removed during the hypnotic state. In a recent medical journal it is reported that a girl underwent an operation while asleep, and being ordered to dress herself, go to bed, and sleep on for an hour, she did so. It is not, however, stated why she should dress herself before going to bed. All this is simply a repetition of what was formerly reported under mesmerism. Nearly half a-century back many operations were performed on the susceptible natives of India by Dr. Esdaile at Madras, who first mesmerised his patients.

Professor Bernheim, of the Faculty of Medicine, Nancy, defines hypnotism as the production of a psychial condition in which the faculty of receiving impressions by suggestion is greatly increased. Although not so common as in years gone by, most of us have seen mesmeric exhibitions when the magnetiser made his victims perform various acts while in the mesmeric trance. One of the latest of such exhibitions was at the Westminster Aquarium some years back, when, if we recollect right, Dr. Donkin questioned the power of the operator, which failed on a new batch of persons from the audience being submitted without selection to the operator's tender mercies. Dr. Tuckey, in his work under notice brings forward various instances tending to evidence the influence of mind and emotion imagination over the body. Such anecdotes need not, however, be repeated. One of the first things a medical student learns is, that there are various nervous maladies which may be excited by the emotions; and practical men are aware that it is in persons predisposed by temperament that such maladies are most readily developed. Moreover we know that mesmerism not unfrequently excited the very maladies it was supposed to cure, such as epileptic convulsions, hysterical manifestations, erotomania, etc., until at last it was said that mesmerism was a means of inducing certain maladies artificially, in order that they might be studied.

According to psychologists two kinds of nervous action go on in the brain the one automatic and instinctive, the other rational, volitional, and deliberative. Hypnotism (like its predecessor, mesmerism) suppresses the latter action, and allows full play to the former. Therefore, the more a man's actions are the result of impulse rather than of reason, the more susceptible he is to external impressions, and therefore to suggestive treatment. But this is not all. There are persons who, when placed artificially asleep, will promise to perform actions when they awake. Although clairvoyance was exploded by the offer of a £500 note if any clairvoyant could name the number and date, still the powers which belonged to the mesmeriser of sometimes being able to make his patients perform acts when they awake remains with the hypnotiser. Only recently a case of stealing a blanket while under hypnotic influence was reported; and another, of stealing a five-franc piece on suggestion.

Hypnotism as an exhibition at public entertainments has been prohibited by law in Switzerland, Holland, and Italy. In Italy it was stated that "it is essential to prevent experiments which, while abolishing consciousness of action, produces morbid effects on predisposed persons, and renders them sub-

* Psycho-Therapeutics; or, Treatment by Sleep and Suggestion. By C. Lloyd Tuckey, M.D. Baillière, Tindal, and Cox, King William Street, Strand. 1889.

ject to the will of others." In a discussion at the New York Academy of Medicine last year it was advanced that however scientifically the subject may be treated, it is so open to abuse that its introduction into general therapeutics might be disastrous. Moreover, we have yet to learn how far hysterical subjects themselves may be permanently affected by the treatment. The dangers of hypnotism are, however, in Dr. Tuckey's opinion, exaggerated, and the stories told of persons obtaining undue influence over others by its means are mostly fables. Still Dr. Tuckey admits that when a person is continually being hypnotised by the same operator the hypnotic state can be produced with surprising readiness. We recollect years ago a mesmeriser asserting the power of mesmerising a susceptible patient many yards away, and even through a brick wall. And in the *British Medical Journal* (page 164, for 1886) it is mentioned that a Mon. Liegeois, of Nancy, sends his clients to sleep by telephone, and then suggests by the same means. We also find it stated that at Bordeaux a personused for public exhibitions became subject to spontaneous sleep, during which he attempted suicide. There are also other instances of persons falling to sleep afterwards, and of impairment of the mental faculties, especially of memory, extending even to a person forgetting his own name. There is also another danger admitted by Dr. Tuckey. He states that in certain cases there arises a craving for the hypnotic sleep, just as there may arise a crave for wine, opium, chloral, or any other stimulant or sedative. It is true that such a craving would not be satisfied by the physician; but as we are told that no special gifts are necessary for an operator, the hysterical craver might obtain satisfaction from elsewhere.

The example of Dr. John Elliottson, once of University College and the North London Hospital, is probably strange to the present generation. This once able physician became a convert to mesmerism, and because he was not permitted to teach and practise this pseudo science in the college and hospital, he resigned his positions. Of course when mesmerism exploded, Dr. Elliottson found his occupation gone, and his professional ruin complete. The hypnotic professors do not certainly affect quite so much as the mesmeric professors of former days, many of whom believed that persons in the mesmeric state—especially women—could see inside themselves, diagnose their disease, and prescribe their remedy. But hypnotic professors believe rather too much if they really think that dyspepsia, chronic alcoholism, and puerperal mania, etc., are to be cured by sleep and suggestion. Doubtless, like the morsel of bread in the gallon of sack, there is some truth in "Psycho-therapeutics, or treatment by sleep and suggestion," and so long as it is used as recommended by Dr. Tuckey, it may probably be used beneficially. Dr. Tuckey says, "The practitioner who uses hypnotism should do so with the same precautions which he adopts in administering an anæsthetic." Moreover, Dr. Tuckey does not altogether disdain to combine medicine with suggestion. Under such conditions, with the evidence before us, it would be unreasonable to doubt that benefit sometimes results to persons who are susceptible to the treatment. On the other hand, it would be equally against evidence to ignore the fact that all persons—probably the majority—are not susceptible; that there are more diseases which would not be benefited than otherwise; that the system is susceptible of abuse; and that it may possibly do injury in one direction while producing an opposite effect in another.

